# A Structural Competency Framework for Emergency Medicine Research: Results from a Scoping Review and Consensus Conference

**DOI:** 10.5811/westjem.2022.6.56056

**Published:** 2022-09-12

**Authors:** Amy Zeidan, Brian Salhi, Anika Backster, Erica Shelton, Alycia Valente, Basmah Safdar, Ambrose Wong, Alessandra Della Porta, Sangil Lee, Todd Schneberk, Jason Wilson, Bjorn Westgard, Margaret Samuels-Kalow

**Affiliations:** * Emory University School of Medicine, Department of Emergency Medicine, Atlanta, Georgia; † Johns Hopkins University School of Medicine, Department of Emergency Medicine, Baltimore, Maryland; ‡ University of Massachusetts, Department of Emergency Medicine, Worcester, Massachusetts; § Yale University, Department of Emergency Medicine, New Haven, Connecticut; ¶ University of Cincinnati, Department of Emergency Medicine, Cincinnati, Ohio; || The University of Iowa Carver College of Medicine, Department of Emergency Medicine, Iowa City, Iowa; # Los Angeles + University of Southern California, Department of Emergency Medicine, Los Angeles, California; ** Morsani College of Medicine, Division of Emergency Medicine, Department of Internal Medicine, Tampa, Florida; †† University of Minnesota Medical School, Department of Emergency Medicine, Minneapolis, Minnesota; ‡‡ Massachusetts General Hospital, Department of Emergency Medicine, Boston, Massachusetts

## Abstract

**Introduction:**

The application of structural competency and structural vulnerability to emergency medicine (EM) research has not been previously described despite EM researchers routinely engaging structurally vulnerable populations. The purpose of this study was to conduct a scoping review and consensus-building process to develop a structurally competent research approach and operational framework relevant to EM research.

**Methods:**

We conducted a scoping review focused on structural competency and structural vulnerability. Results of the review informed the development of a structural competency research framework that was presented throughout a multi-step consensus process culminating in the 2021 Society for Academic Emergency Medicine Consensus Conference. Feedback to the framework was incorporated throughout the conference.

**Results:**

The scoping review produced 291 articles with 123 articles relevant to EM research. All 123 articles underwent full-text review and data extraction following a standardized data extraction form. Most of the articles acknowledged or described structures that lead to inequities with a variety of methodological approaches used to operationalize structural competency and/or structural vulnerability. The framework developed aligned with components of the research process, drawing upon methodologies from studies included in the scoping review.

**Conclusion:**

The framework developed provides a starting point for EM researchers seeking to understand, acknowledge, and incorporate structural competency into EM research. By incorporating components of the framework, researchers may enhance their ability to address social, historical, political, and economic forces that lead to health inequities, reframing drivers of inequities away from individual factors and focusing on structural factors.

## INTRODUCTION

The emergency department (ED) has long been recognized as a “safety net” of the United States’ healthcare system, serving as a portal of entry for people and communities that would otherwise be unable to access care.^1^ Much of the difficulty in accessing care results from structural inequities and barriers faced by these populations (eg, lack of health insurance, paid leave, transportation) rather than personal choice or preference.^2^ The ED, therefore, serves as critical setting to examine and address structural barriers to care, “upstream” drivers of health-seeking behaviors, and contributors to health inequities.

Recent trends in “social emergency medicine” (EM) have made strides to reframe the healthcare encounter in structural—rather than individualistic—terms.^3^,^4^,^5^ We take social EM to refer to a general *approach* to understanding how historical, political, and economic conditions impact health, disease, and the practice of EM. Importantly, this approach is undertaken to promote conditions and practices that may lead to a more equitable and, therefore, healthier society. In other words, we conceive of social EM as relevant to all EM research topical areas (eg, cardiovascular care, trauma) rather than comprising a distinct or unrelated topical area.

Despite the clear relevance of this approach to EM research and practice, there is limited literature addressing *how* to incorporate such an approach in EM, especially within the research process. To address this gap in the literature, we drew on the theoretical framework of structural competency, which is defined as the trained ability for health professionals to recognize and respond to signs and symptoms of individual illness as the downstream effects of broad historical, social, political, and economic structures.^3^ Throughout this paper, we use “structure” to refer to the ways that society is hierarchically organized through institutions, political and economic policies, and normative beliefs—thus beyond the powers of an individual actor to overcome, change, or reform. Structural competency was first conceived as a framework to inform medical education and has been used to develop educational curricula and clinical tools for learners at all stages in medical training.^6^,^7^,^8^

We argue that the structural competency framework may be extended from education to research in the ED setting, especially when coupled with a related term, “structural vulnerability.” Structural vulnerability refers to physical and emotional suffering among specific groups and individuals that results or is made worse from patterns of bias and advantage/disadvantage across organizations, institutions, governments, and social networks.^9^,^10^ This suffering is resultant from or exacerbated by class-based economic exploitation and cultural, gender/sexual, racialized, and other forms of discrimination, rather than individual actions or “choices.”^8^ While structural competency and structural vulnerability are related to social determinants of health, defined as conditions in one’s environment that impact their health and health outcomes, they are distinct in their focus on how political decisions, economic systems, and historical context produce social determinants of health (eg, differential access to material goods and opportunities).^11^,^3^,^8^ Application of structural competency and structural vulnerability to EM research is paramount given our specialty’s growing calls to address and redress structural and health inequities.^12^ Creating a framework for structurally competent research within EM is a critical step in moving toward EM research that is inclusive and collaborative, and accounts for the historical and structural forces that impact healthcare delivery and health outcomes.

To apply concepts of structural competency and structural vulnerability to EM research, we conducted a scoping review of structural competency and structural vulnerability literature and engaged in a multi-step consensus process culminating in the 2021 Consensus Conference of the Society for Academic Emergency Medicine (SAEM). In this paper we report findings from the scoping review and consensus conference, providing a theoretical framework to incorporate structural competency concepts in the EM research process.

## MATERIALS AND METHODS

### Scoping Review

We conducted a scoping review of published work focused on structural competency and structural vulnerability following Arksey and O’Malley’s six-step framework for scoping reviews, with the exception of the optional consultation exercise.^13^ Our aims were to 1) provide a comprehensive overview of literature published on structural competency and structural vulnerability; 2) identify the ways in which structural competency and structural vulnerability have been operationalized in published research; 3) identify existing gaps in the literature that could inform future research in EM; and 4) identify methodological approaches salient to EM research.

We identified relevant studies using the key terms “structural vulnerability” and “structural competency” searching records published before November 2020 in MEDLINE, Scopus, and Web of Science. All publication types (eg, original research, reviews, perspectives) and methods (qualitative and quantitative) were considered. Articles were included in the initial screen if they were published in English, performed in the US or Canada, and addressed a topic broadly relevant to EM. The remaining articles were reviewed by two independent reviewers for title and abstract screening and inclusion to determine whether the articles were relevant to EM research or education. Any disagreement between the independent reviewers was resolved by BAS and AZ. Eligible articles were reviewed by two additional independent reviewers, who used Covidence^14^ to complete a standardized data extraction form developed a priori (Table 1). Extracted variables included literature characteristics and free-text variables related to study aims.

### Consensus Building Process

The scoping review was undertaken alongside a multi-step consensus process culminating in the 2021 SAEM Consensus Conference, which aimed to create a focused research agenda for social EM and population health.^15^ Briefly, the consensus-building process began in the year prior to the SAEM meeting and included working groups that met regularly to discuss findings from the scoping review, develop a structural competency framework for EM research, and to shape content for two conference breakout sessions. During the breakout sessions, the working group leaders (BAS and AZ) presented an assessment of the current literature and a draft of the research framework to operationalize the concepts of structural competency and structural vulnerability. Attendees included SAEM members and non-SAEM stakeholders, all of whom provided feedback during breakout sessions and participated in anonymous surveys following each session.

### Development of the Research Framework

Results from the scoping review and feedback from the consensus-building process were used to develop an operational framework for applying structural competency to EM research. The following objectives were considered when developing the framework: 1) acknowledgment of structural forces, structural vulnerabilities, and systemic causes of health inequities and how these impact patients, their health-seeking behaviors, ability to pursue treatment plans, and health outcomes; 2) consideration of how systemic causes of health inequities impact an individual’s involvement in research, specifically recognizing the long and ongoing legacy of injustice and exploitation in medical research; and 3) operationalization of structural competency throughout the research process including study purpose, study design, data collection, data analysis, and dissemination. We recognize that there is significant variability in research questions, methods, and analysis and have, therefore, designed the framework to be incorporated in part, or in whole, as deemed appropriate by researchers.

## RESULTS

### Scoping Review Results

The literature review produced 291 articles of which 123 articles were determined relevant to EM research and 51 relevant to EM education after title and abstract review. All articles underwent full text review and data extraction following the standardized data extraction form (see Table 2). (Results from the education review are presented elsewhere).^16^ Most articles were published in public health, sociology and anthropology, or multidisciplinary journals, and the majority of articles represented original research (predominantly ethnographic and interview study designs). Only three studies were conducted directly in the ED or focused on ED populations^17^,^18^,^19^; none of the studies were published in EM journals. For studies that were original research, 48% (n = 47) included community partners,^20^,^21^,^22^ and 17% of studies (n = 18) included the study population (see Appendix for examples). 23,24,25,26 Nearly half of the articles explicitly defined structural competency (40%) or structural vulnerability (15%), and most articles acknowledged or described structures or systems that lead to inequities (85%).

Articles included in this review were not characterized by a specific population or single topical area of interest. For example, populations examined migrant workers, sex workers, people who use^27^ drugs, people living with human immunodeficiency virus/sexually transmitted infections, people experiencing homelessness, incarcerated people, LGBTQI communities, racialized populations, and communities disproportionally affected by COVID-19. Analytical and explanatory models within these articles, therefore, shifted responsibility away from the individual and toward the system in which a person or community is living (ie, structural competency). Papers described and analyzed how health and social outcomes of communities were resultant from their place in social, political, cultural, and economic hierarchies determined by complex power structures that often reinforce subordinated status (ie, structural vulnerability). Researchers also drew upon a related concept, “structural violence,” which refers to the ways in which structures of power render some people “unable to achieve their capacities or capabilities to their full potential, and almost certainly if they are unable to do so to the same extent as others.”^27^

### Consensus Conference Feedback

Feedback from the first breakout session highlighted the difficulty of defining the “community,” specifically who is a part of the community or study population, who may be appropriate to represent the study population, and how to define the role of community advisors/partners. Much of the discussion focused on community-based participatory research (CBPR) and incorporating or distinguishing this methodology within the structural competency framework. Overall, participants determined that CBPR may not be applicable to all EM research, whereas the structural competency framework is meant to be used in all types of EM research. Participants also emphasized the need to center the needs of the study population when developing the research question, which might be accomplished by engaging the study population prior to the start of the study.

During the second consensus conference session, participants discussed how to operationalize the needs of the community within the research framework, specifically recommending that community needs be identified prior to the start of the study, as well as incorporating existing efforts within that community. Respondents also suggested that, given histories of structural vulnerability, research teams should focus on strengths rather than focusing only on deficiencies among study populations. Finally, participants discussed that a framework that foregrounds structural competency must also consider the asymmetries and inequities that are manifest in regulatory structures, including institutional review boards (IRB), as well as funding institutions and pipelines.

### A Structurally Competent Research Approach and Operational Framework

Results of the scoping review informed the development of a structurally competent research approach and framework. The framework was specifically created in alignment with components of the research process including the following: 1) defining the research question; 2) study design; 3) data collection; 4) data analysis/interpretation; and 5) dissemination. Feedback from the Consensus Conference was incorporated to modify and refine the framework. We detail specific examples in the following section and provide a visual depiction in Table 3. Using specific examples from the articles reviewed, the following section describes a structurally competent research framework. This framework empowers EM researchers to understand, acknowledge, and take into account structural forces and barriers impacting ED patients, and to act ethically in carrying out research and intervening at system and community levels to maximize patient health outcomes.

#### Defining the Research Question and/or Study Purpose

Developing a well-considered research question and/or study purpose is the cornerstone of valid, impactful research. It is, therefore, critical that EM researchers examine their research question for implicit assumptions that may influence the methods and analysis. We advocate that the literature reviewed for the study background draw on existing work from related disciplines, including history, sociology, and anthropology (among others) and to reconsider the research question in light of this evidence. Ideally. and if applicable, the research question should incorporate or acknowledge the impact of structural forces on the proposed study population(s). Whenever possible, the study population may be engaged in the initial stages to assist in developing a research question and potential outcomes that address their priorities and recognize their strengths and vulnerabilities to ensure that the research question aligns with their interests.^28^

For example, Kolla and Strike^21^ provide a salient example of this approach in their examination of the structural vulnerabilities of harm-reduction workers and people who use drugs in an overdose education and naloxone distribution (OEND) program. While noting that OEND programs have made major strides toward preventing overdose deaths, they extend their research question beyond relative risk reduction of naloxone provision and note the ongoing structural limitations and unintended side effects of these programs. For example, the authors cite examples of criminalization and stigma applied to those who use drugs, thereby exacerbating barriers to seeking help (eg, police accompanying ambulances for overdose response, which exacerbates fears and limits access to healthcare services). In this example, the authors contextualize the research question within the historical and political examples relevant to their specific study populations. While this study was not conducted in an ED setting people who use drugs frequently receive care in the ED—often as a direct result of stigma and criminalization associated with drug use. Taking these histories and vulnerabilities into account is, therefore, critical to asking insightful and impactful EM research questions.

#### Study Design

In developing the research design, it is important to consider the study population’s relationships with the healthcare system, historical research practices, and/or the researchers’ institutions writ large. Taking the time to consider these factors provides important insights for the study design, including best practices for recruitment, consent, incentive, and implementation of an intervention (depending on the study design). If applicable, the study team could consider developing relationships with the target population to better understand their experiences and/or partnering with study participants or representatives of the study community (community advisory board, community partners, stakeholders, proxies, etc.) to develop the design. While the term “community” may have a variety of interpretations and definitions, we encourage the study team to consider groups or organizations that are representative and inclusive of the study population, incorporating suggestions from individuals with lived experiences relevant to the study population whenever possible. Relatedly, it is important to remember that single individuals acting as community representatives may not successfully represent all perspectives of the community. We stress that relationships with community partners and other stakeholders be longitudinal to the degree that it is possible and/or appropriate. Partnerships that are forged solely for the sake of research purposes may be perceived as extractive or exploitative, therefore perpetuating harms and distrust.

For example, Cheney et al^29^ used a formalized community-based participatory research (CBPR) approach to develop sustainable partnerships with local farmworkers and farmworker organizations in studying how poverty and inequality affect the health of foreign-born Latinos. Prior to the start of the study, the study team engaged community leaders, advocacy groups, farmworkers, healthcare clinicians, and political officials to understand the community needs and research capacity, and to explore potentially salient research topics. This allowed for the study team to define a research question relevant to the community (eg, alcohol use among farmworkers), and engage them throughout the research process—including the development of the research question, study design, recruitment, data analysis and interpretation, and dissemination of findings.

Notably, CBPR is a methodological approach that considers historical, economic, and political contexts and engages community members as partners in the research process to develop trust and community capacity to engage in research.^30^,^31^ Community-based participatory research is a well-established and valuable research methodology but may not be possible to carry out in all research contexts. Like CBPR, we prioritize consideration of historical, economic, and political contexts and engagement of community members whenever possible. However, we also argue that attention to structural forces and processes is paramount even when community engagement is not feasible or applicable.

For example, Willging et al^32^ incorporate frameworks of structural competency and vulnerability in their interview-based study of transgender and gender non-conforming (TGGNC) ED patients. The authors describe how TGGNC patients are often denied social services, which in turn exacerbates structural vulnerabilities (ie, access to medical and social services, unemployment, housing instability, violence/trauma) and places them at risk of adverse health outcomes. Participants in the study described unstable employment and economic challenges as a barrier to insurance and, thus, access to care beyond the ED. They also described an increased risk of violence and physical injury related to stigma and discrimination, which is often addressed and treated in the ED. The authors effectively incorporate a structural competency framework (using a non-CBPR methodology) to highlight the structural issues that adversely impact the health and wellbeing of TGGNC ED patients. Moreover, the authors extend their findings to help address contributors to delayed care and to suggest structurally competent services for TGGNC patients.

We stress that frameworks of structural competency and vulnerability are not limited to qualitative studies or the social sciences, despite their predominance in this review. For example, for studies that rely on large datasets, EM researchers can still be cognizant of the ways that data is collected (eg, questions asked, language of questionnaires, etc.) and whether the study methodology may overlook or perpetuate inequities in care.

#### Data Collection and Storage

In addition to following IRB guidelines, extra consideration may be helpful to ensure participants feel valued and respected during the data collection process and to mitigate any power differentials that may discourage participation or quality data collection. Researchers should, for example, ask: Who is collecting the data? What is the setting of recruitment or engagement and how may this affect data collection? How is data being collected (eg, written vs electronic)? What is the most appropriate language of data collection (eg, should the study team include a bilingual member)? Who will have access to the data during the study and at the completion of the study?

For example, Organista et al^26^ studied the relationship between psychological distress and alcohol and substance-related sexual risk in Latino migrant day laborers. Recognizing the stigma associated with their study topic and the power differential between the research team and the study population, Organista et al included an “expert informant,” a day laborer from the study population, and partnered directly with the San Francisco Day Labor Program, a local community organization, to engage participants. To ensure ethical engagement and quality data collection, recruitment occurred at the community partner site, interviews were conducted in the participant’s language, and some interviews were completed directly by the expert informant.

It is important to pay particular attention to challenges with anonymity and data protection when conducting qualitative research, where individuals’ stories, experiences, and voices are the central focus of data collection. Researchers should be attuned that vulnerability may be especially heightened among ED patients and should explore options to mitigate these vulnerabilities.

#### Data Analysis and Interpretation

In analyzing the data and applying results to future practices and policies, it may be helpful to consider in advance how the data will be used, who will be reviewing the data and providing feedback (ie, the study population or community representatives), how results could impact the study population and whether results are interpreted with respect to existing structural forces and structural vulnerabilities of the study population. Similarly, it may be helpful to discuss what outcomes are important to the study population/stakeholders, particularly if these outcomes differ from those of the research team. It may also be important to consider demographic factors and how they are interpreted or rather misinterpreted as “risk factors” rather than structural vulnerabilities. Indeed, when racial and ethnic health inequities are found, we urge that researchers, reviewers, editors, and readers ask *why* and *how* these come to be.^33^ Frameworks of structural competency and vulnerability are especially useful in highlighting how health inequities are produced without resorting to fallacies of biological difference.

In the study by Mayer et al^25^ of patients’ motivations to initiate injectable hydromorphone and diacetylmorphine treatment, preliminary findings were reviewed by a community advisory board that consisted of representatives from the target population. Results were also contextualized within the target population’s structural vulnerabilities, including poverty, food insecurity, housing insecurity, criminalization, and how these vulnerabilities influenced their experiences when initiating treatment for opioid use disorder. The authors demonstrate that understanding the social context and existing structural vulnerabilities of the study population are imperative when considering successful treatment initiation.

#### Dissemination

Dissemination is critical to maximizing the impact of research and shaping future research questions. Researchers in EM should consider disseminating results beyond an EM audience to include multidisciplinary and open-access options. Because research findings are often not readily accessible to participants or the broader public (eg, due to costs and/or technical language), researchers should consider alternate mechanisms for disseminating findings back to the target population and broader public (eg, local news, podcasts, healthcare institutions, varying levels of government) to model transparency and engender trust in research.

## DISCUSSION

Results of the scoping review and development of the framework described here provide an opportunity for EM researchers to incorporate concepts of structural competency and structural vulnerability in EM research. As the ED continues to serve as a safety net for structurally vulnerable populations, we are uniquely positioned to address conditions of suffering and contributors to poor health. By incorporating frameworks of structural competency and vulnerability, we may be better equipped to recognize and address the health inequities and the complexities of ED care.

Relatedly, Metzl et al^34^ describe a structurally competent research agenda specific to firearm and mental health research, specifically focused on how to study mass shootings and multiple-victim gun homicides. The authors consider contextual factors of gun policies and laws (eg, inaccurate narratives that people with mental illness are more dangerous, resulting in legislation requiring mental health professionals to report patients who pose a “risk”), the racialization of gun violence, community over policing, and the meaning and value that different individuals ascribe to guns. In considering these myriad contributors to gun violence in the US, the authors provide a research framework that incorporates structural interventions, antiracist gun research, messaging from trusted sources, and politically neutral policies that focus on violence prevention. The authors advocate that this approach will ensure research targets effective and equitable interventions and policies for all people and communities. Metzl and colleagues provide an example of how to apply structural components to a topic-specific research agenda, in this case firearm injury and mental health, that could be applied to other EM-relevant research topics.

The proposed framework allows EM researchers to build on the aforementioned examples to directly apply structural competency and vulnerability to research principles and processes, for example, by redefining the study question through acknowledgment of existing and historical structures and systems, collaborating with the study population and community partners that serve the study population, and analyzing data using a structural vulnerability lens.

## LIMITATIONS

Our review included only studies that used frameworks of structural competency and structural vulnerability. Our search terms may have excluded published papers that add to our understanding of the ways that historical, political, and economic structures influence health, illness, clinical care delivery, and their related research. However, our database search results underwent multiple reviews and discussions, and we are confident that the data presented are representative of the current state of structural competency and structural vulnerability and their applicability to EM research. Second, we excluded studies published outside the US or Canada. While we recognize that historical, political, and economic structures are salient to EM research across the world, we sought to summarize data and propose a framework adaptable to EM research in the US and Canada, and we believe that our selection criteria have accomplished this. Finally, the transferability of findings to EM research may be limited by the small number of articles conducted in an EM setting. Nevertheless, a large body of evidence strongly suggests that EM research is fertile ground for a unifying structural competency framework.^12^

## CONCLUSION

Since its inception, EM has interfaced with a broad spectrum of patients, especially those with structural vulnerabilities. A growing body of EM research has focused on upstream drivers of ED patients’ presentations and health outcomes. Our scoping review and structurally competent research framework outline considerations and tangible strategies for engaging structurally vulnerable populations and making strides to eliminate health inequities.

## Supplementary Information



## Figures and Tables

**Table 1:** Scoping review data-extraction form.

Article characteristics	Study title Journal name Year published Funded (yes/no, if yes, source) Publication/article type (Letter to the editor; Editorial/Commentary; Case study/case report; review, Original research; Other) Study type (Experimental study; RCT; Cohort study; Observational study; Survey; Focus group and/or interview study; Ethnographic study; Community-based research; Other) Academic discipline of journal (Undergraduate ME; Emergency Medicine; Psychiatry/Psychology/Mental Health; Primary Care; Infectious Disease; Sociology; Anthropology; Nursing; Social Work; Public Health; Other or Multidisciplinary)
Research-related variables	Research question/Purpose (free text) Topic/Category – choose all that apply (Community Health; COVID-19 pandemic; Food insecurity; Gender disparities; HIV/STI; Homelessness; Immigration; Incarceration/Policing; LGBTQ+; Mental Health; Migrant or Farm Labor; Race/Racial disparities; Sex work; Substance use, Violence; Other/Free text) Inclusion Criteria (free text, not explicitly described) Exclusion Criteria (free text, not explicitly described) Study population: sex, gender, race/ethnicity, language, subpopulation (free text) Inclusion of community partners on research team or with research protocol? (yes/no) ○ If yes, describe in free text Inclusion of study population on research team or with research protocol? (yes/no) ○ If yes, describe in free text Recruitment process/methods ○ Direct recruitment of participants through community organization/partner; Direct recruitment in a healthcare setting; Direct recruitment of participants known to study team; Solicitation of participation through advertisements/media notices/community flyers. ○ Other: Free text ○ Not applicable Consent process ○ written/verbal/waived/community consent/mixed ○ other/free text/interpretation present/translation used for consent Incentive ○ yes/no ○ If yes, type of incentive: direct cash payment; gift card or voucher; gift/good exchange; other: free text. Intervention ○ yes/no/not applicable ○ If yes, describe via free text Outcome Measures: (free text or not applicable)
Structural competency related variables	Was structural competency defined? ○ yes/no/other ○ If yes, describe how structural competency was defined (free text) How was structural competency operationalized? ○ Acknowledgment/description of structures/systems that lead to inequities? (Single issue SDH-related component vs broader structural competency) Other observations/notes

*RCT*, randomized control trial; *ME*, medical education; *COVID-19*, coronavirus disease 2019; *HIV*, human immunodeficiency virus; *STI*, sexually transmitted infection; *LGBTQ+*, lesbian, gay, bisexual, transgender, queer/questioning+; *SDH*, social determinants of health.

**Table 2:** Scoping review article characteristics.

	n
Academic discipline	
Sociology or Anthropology	36
Public health	33
Multidisciplinary	20
Psychiatry, psychology, or mental health	7
Infectious disease	6
Policy	5
Substance use	4
Public policy	2
Palliative care	2
Social work	2
Drug policy	2
Primary care and Public health	1
Nursing	1
Population health	1
Primary care	1
Publication type	
Case study/Case report	3
Editorial/Commentary	13
Original Research	104
Letter to Editor	1
Other	2
Study design	
Interview study	35
Ethnographic study	26
Mixed design	17
Not applicable (e.g., opinion piece, letter to editor)	14
Survey study	9
Observational study	5
Community-based research	3
Evidence review	3
Systematic review	3
Cohort study	3
Focus group	2
Experimental study	1
Non-randomized experimental study	1
Inclusion of community partners	
Yes	47
No	51
N/A	25
Inclusion of study population	
Yes	18
No	85
N/A	20
Recruitment process	
Not applicable	29
Direct recruitment through community partners	28
Direct recruitment of participants known to study team	23
Direct recruitment through healthcare setting	13
Mixed	11
Targeted population	9
Canvassing	8
Direct referral	2
Was structural competency defined?	
Yes	49
No	47
N/A	27
How was structural competency operationalized?	
Acknowledgment/description of the structures or systems that lead to inequities	104
N/A or not operationalized	10
Reference to single-issue social determinant of health (e.g., homelessness)	5
Other	5

*N/A*, not applicable.

**Table 3:** Structural competency framework recommendations.

Research phase	Description	Checklist of recommended actions	Key sample references
Phase 1: Defining the Research Question	Study team examines research question for implicit assumptions and incorporates structural forces and structural vulnerabilities of the study population	▪ Does the literature review incorporate structural vulnerabilities of study population(s)? ▪ Does the research question acknowledge the impact of structural forces (historical, social, political, and economic structures) and how this has led to health inequities of study populations? ▪ Has the study team engaged with study populations/communities when defining the research question? ▪ Does the research team include members from the study populations/representative community members who provide input regarding the study question? ▪ Does the background work incorporate strengths of study populations and key works from researchers/community organizations representing the study populations?	Holmes SM. “Is it worth risking your life?”: Ethnography, risk, and death on the U.S.-Mexico border. Social Science and Medicine. 2013;99:153–6 Kolla G, Strike C. ‘It’s too much, I’m getting really tired of it’: Overdose response and structural vulnerabilities among harm reduction workers in community settings. International Journal of Drug Policy. 2019;74:127–35
Phase 2: Study Design	Study team incorporates structurally sensitive elements into study design and uses ideal processes to involve study populations	▪ How have the study populations historically interacted with the health system? Does the design account for how the study populations may been negatively impacted by medical research? ▪ Does the study team have a prior relationship with the study populations/representative community members or community organizations? If not, consider revisiting Phase 1 to develop meaningful partnerships and implore community-based participatory research (CBPR). ▪ If appropriate for the study design, employ CBPR and recruit those familiar with this methodology. ▪ Inclusion/Exclusion Criteria: Does the criteria unintentionally exclude specific populations (eg, language requirement, insurance status, etc)? ▪ Recruitment Process: Where are subjects recruited, who is recruiting subjects, will subjects feel comfortable with the recruitment location and study team member recruiting? ▪ Consent process: Is consent equally available to all study populations? Who is providing consent, and will study populations feel comfortable with the consent process? Will written consent be a barrier for participation? ▪ Incentive: Is the form of incentive accessible to all study populations and free of bias?	Wilmsen C. Working in the Shadows: Safety and Health in Forestry Services in Southern Oregon. J Forest 2015;113(3):315–24. Cheney AM, Newkirk C, Rodriguez K, Montez A. Inequality, and health among foreign-born Latinos in rural borderland communities. Social Science and Medicine. 2018:115–22.
Phase 3: Data Collection/Storage	Study team recognizes ideal methods for data collection and storage that recognize and mitigate structural forces	▪ Who will be collecting the data? Will study populations feel comfortable with the individuals collecting the data? ▪ How is data being collected (written vs electronic), in what language, and is this the ideal method for data collection? ▪ How will data be stored, and will appropriate individuals have access to data? Will data be stored at a community site, hospital site, etc?	Organista KC, Arreola SG, Neilands TB. La desesperación in Latino migrant day laborers and its role in alcohol and substance-related sexual risk. SSM - Population Health. 2016;2:32–42.
Phase 4: Data Analysis/Interpretation	Study team members analyzing data consider context, feedback, and implications of results	▪ Is data analyzed within the context of structural vulnerabilities of the study population? ▪ Are appropriate members of the study team involved in analysis/interpretation, specifically those with lived experience representing the study populations? ▪ Who will be providing feedback regarding data analysis, and how will feedback be incorporated? ▪ How may results impact the study populations negatively or positively? ▪ How will this data be used? What are the implications of the results?	Mayer S, Fowler A, Brohman I, et al. Motivations to initiate injectable hydromorphone and diacetylmorphine treatment: a qualitative study of patient experiences in Vancouver, Canada. International Journal of Drug Policy. 2020;85:102930
Phase 5: Dissemination/Policy Change	Study team employs unique strategies for dissemination and incorporates opportunities for policy change	▪ Consider dissemination of results beyond EM audience targeting multidisciplinary sources and avenues other than academic publications. ▪ When possible, opt for open access for publications. ▪ Determine mechanism to disseminate findings to study populations. ▪ Consider how results will be translated to policy change.	

*EM*, emergency medicine.
