# Supplementary material for: A Structural Competency Framework for Emergency Medicine Research: Results from a Scoping Review and Consensus Conference
Source: West J Emerg Med. 2022 Sep 12;23(5):650–9. doi: 10.5811/westjem.2022.6.56056 (PMC9541992; doi:10.5811/westjem.2022.6.56056)
Supplement: Supplementary file 1 [file wjem-23-650-s001.docx]

Appendix A: Inclusion of Community Partners and Study Population Selected References

| **Data Extraction Variable** | **Summary** | **References** |
| --- | --- | --- |
| Inclusion of Community Partners | Common methodological approaches included use of an advisory committee with patient representatives, new or existing partnerships with community organizations serving the targeted population, and carrying out study components at the site of the community partner (ex. Recruitment, focus groups, interviews) | 1. Krüsi A, Kerr T, Taylor C, Rhodes T, Shannon K. “They won’t change it back in their heads that we’re trash”: the intersection of sex work-related stigma and evolving policing strategies. Sociology of health & illness 2016;38(7):1137–50.  2. Logan RI. ‘A poverty in understanding’: Assessing the structural challenges experienced by community health workers and their clients. Global Public Health 2020;15(1):137–50.  3. Kolla G, Strike C. ‘It’s too much, I’m getting really tired of it’: Overdose response and structural vulnerabilities among harm reduction workers in community settings. International Journal of Drug Policy 2019;74:127–35.  4. Kelley RI, Ivey SL, Silver K, Holmes SM. “If We Don’t Produce, Bring Another:” Work Organization and Tomato Worker Health. Journal of Agromedicine 2020;25(3):286–301.  5. Stajduhar KI, Mollison A, Giesbrecht M, et al. “Just too busy living in the moment and surviving”: barriers to accessing health care for structurally vulnerable populations at end-of-life. BMC Palliative Care 2019;18(1):1–14.  6. LeBrón AMW, Schulz AJ, Gamboa C, Reyes A, Viruell-Fuentes EA, Israel BA. “They Are Clipping Our Wings”: Health Implications of Restrictive Immigrant Policies for Mexican-Origin Women in a Northern Border Community. Race and Social Problems 2018;10(3):174–92.  7. Kenny KS, Barrington C, Green SL. “I felt for a long time like everything beautiful in me had been taken out”: Women’s suffering, remembering, and survival following the loss of child custody. International Journal of Drug Policy 2015;26(11):1158–66.  8. Andress L, Hall T, Davis S, Levine J, Cripps K, Guinn D. Addressing power dynamics in community-engaged research partnerships. Journal of Patient-Reported Outcomes 2020;4(1):0–7.  9. Jenkins E, Haines-Saah R, McGuinness L, et al. Assessing the impacts of the Agenda Gap intervention for youth mental health promotion through policy engagement: A study protocol. International Journal of Mental Health Systems 2020;14(1):1–11.  10. von Glascoe CA, Schwartz NA. Bad lungs bad air: Childhood asthma and ecosyndemics among Mexican immigrant farmworkers of California’s San Joaquin Valley. Human Organization 2019;78(2):110–21.  11. Silberzahn BE, Morris MB, Riegger KE, et al. Barriers and facilitators to retaining a cohort of street-based cisgender female sex workers recruited in Baltimore, Maryland, USA: Results from the SAPPHIRE study. BMC Public Health 2020;20(1):1–12.  12. Stajduhar KI, Giesbrecht M, Mollison A, Dosani N, McNeil R. Caregiving at the margins: An ethnographic exploration of family caregivers experiences providing care for structurally vulnerable populations at the end-of-life. Palliative Medicine 2020;34(7):946–53.  13. Fleming MD, Shim JK, Yen I, Van Natta M, Hanssmann C, Burke NJ. Caring for “Super-utilizers”: Neoliberal Social Assistance in the Safety-net. Medical Anthropology Quarterly 2019;33(2):173–90.  14. Quandt SA, LaMonto NJ, Mora DC, Talton JW, Laurienti PJ, Arcury TA. COVID-19 pandemic among latinx farmworker and non-farmworker families in North Carolina: Knowledge, risk perceptions, and preventive behaviors. International Journal of Environmental Research and Public Health 2020;17(5786):1–17.  15. Freeman R, Gwadz MV, Silverman E, et al. Critical race theory as a tool for understanding poor engagement along the HIV care continuum among African American/Black and Hispanic persons living with HIV in the United States: A qualitative exploration. International Journal for Equity in Health 2017;16(1):1–14.  16. Summers P, Quandt SA, Talton JW, Galván L, Arcury TA. Hidden farmworker labor camps in North Carolina: An indicator of structural vulnerability. American Journal of Public Health 2015;105(12):2570–5.  17. Quandt SA, Arnold TJ, Mora DC, Arcury TA, Talton JW, Daniel SS. Hired Latinx Child Farmworkers in North Carolina: Educational Status and Experience Through a Social Justice Lens. New Solutions 2021;30(4):282–93.  18. Giesbrecht M, Stajduhar KI, Mollison A, et al. Hospitals, clinics, and palliative care units: Place-based experiences of formal healthcare settings by people experiencing structural vulnerability at the end-of-life. Health and Place 2018;53:43–51.  19. Brantley ML, Kerrigan D, German D, Lim S SS. Identifying patterns of social and economic hardship among structurally vulnerable women: a latent class analysis of HIV/STI risk. AIDS Behavior 2017;21(10):3047–56.  20. Miller CL, Fielden SJ, Tyndall MW, Zhang R, Gipson K SK. Individual and structural vulnerability among female youth who exchange sex for survival. Journal of Adolscent Health 2011;49(1):1–7.  21. Cheney AM, Newkirk C, Rodriguez K, Montez A. Inequality and health among foreign-born latinos in rural borderland communities. Social Science and Medicine 2018;215:115–22.  22. Friedman J, Syvertsen JL, Bourgois P, Bui A, Beletsky L, Pollini R. Intersectional structural vulnerability to abusive policing among people who inject drugs: A mixed methods assessment in california’s central valley. International Journal of Drug Policy 2021;87:102981.  23. Organista KC, Arreola SG, Neilands TB. La desesperación in Latino migrant day laborers and its role in alcohol and substance-related sexual risk. SSM - Population Health 2016;2:32–42.  24. Fernández-Esquer ME, Agoff MC, Leal IM. Living Sin Papeles: Undocumented Latino Workers Negotiating Life in “Illegality.” Hispanic Journal of Behavioral Sciences 2017;39(1):3–18.  25. Mayer S, Fowler A, Brohman I, et al. Motivations to initiate injectable hydromorphone and diacetylmorphine treatment: A qualitative study of patient experiences in Vancouver, Canada. International Journal of Drug Policy 2020;85(September):102930.  26. McNeil R, Kerr T, Anderson S, Maher L, Keewatin C, Milloy MJ, Wood E SW. Negotiating structural vulnerability following regulatory changes to a provincial methadone program in Vancouver, Canada: A qualitative study. Social Science and Medicine 2015;133:168–76.  27. Duke MR. Neocolonialism and Health Care Access among Marshall Islanders in the United States. Medical Anthropology Quarterly 2017;31(3):422–39.  28. Quesada J. No soy welferero: Undocumented latino laborers in the crosshairs of legitimation maneuvers. Medical Anthropology: Cross Cultural Studies in Health and Illness 2011;30(4):386–408.  29. Tulimiero M, Garcia M, Rodriguez M, Cheney AM. Overcoming Barriers to Health Care Access in Rural Latino Communities: An Innovative Model in the Eastern Coachella Valley. Journal of Rural Health 2020;00:1–10.  30. Kline N. Pathogenic Policy: Immigrant Policing, Fear, and Parallel Medical Systems in the US South. Medical Anthropology: Cross Cultural Studies in Health and Illness 2017;36(4):396–410.  31. Boyd S, Ivsins A, Murray D. Problematizing the DSM-5 criteria for opioid use disorder: A qualitative analysis. International Journal of Drug Policy 2020;78:102690.  32. Decker MR, Marshall B, Emereson M, Kalamar A, Covarrubias L, Astone N, Wang Z GE. Respondent-driven sampling for an adolescent health study in vulnerable urban settings: a multi-country study. Journal of Adolscent Health 2014;55(6 0):1–17.  33. Snell-rood C, Staton M, Kheibari A. Rural women’s first person perspectives on the role of mental health in substance use. Rural and Remote Health 2019;19(4):5279.  34. Organista KC, Worby PA, Quesada J, Arreola SG, Kral AH KS. Sexual Health of Latino Migrant Day Labourers under Conditions of Structural Vulnerability. Culture, Health and Sexuality 2013;15(1):1–14.  35. Wallace B, Barber K, Pauly B (Bernie). Sheltering risks: Implementation of harm reduction in homeless shelters during an overdose emergency. International Journal of Drug Policy 2018;53:83–9.  36. White RH, Park JN, Galai N, et al. Short-term interruptions to sex work among a prospective cohort of street-based cisgender female sex workers in Baltimore. International Journal of Drug Policy 2020;84:102858.  37. Knight KR, Lopez AM, Comfort M, Shumway M, Cohen J RE. Single Room Occupancy (SRO) hotels as mental health risk environments among impoverished women: the intersection of policy, drug use, trauma, and urban space. International Journal of Drug Policy 204AD;25(3):556–61.  38. Natta M Van, Burke NJ, Yen IH, et al. Stratified citizenship, stratified health: Examining latinx legal status in the U.S. healthcare safety net. Social Science and Medicine 2019;220:49–55.  39. Willging C, Gunderson L, Shattuck D, Sturm R, Lawyer A, Crandall C. Structural competency in emergency medicine services for transgender and gender non-conforming patients. Social Science and Medicine 2019;222(July 2018):67–75.  40. Glick JL, Lim S, Lim S, et al. Structural vulnerabilities and HIV risk among sexual minority female sex workers (SM-FSW) by identity and behavior in Baltimore, MD. Harm Reduction Journal 2020;17(1):1–9.  41. Valdez ES, Valdez LA, Sabo S. Structural vulnerability among migrating women and children fleeing central america and Mexico: The public health impact of “humanitarian parole.” Frontiers in Public Health 2015;3(163):1–8.  42. Worby PA, Organista KC, Kral AH, Quesada J, Arreola S, Khoury S. Structural vulnerability and problem drinking among Latino Migrant day laborers in the San Francisco Bay Area. Journal of Health Care for the Poor and Underserved 2014;25(3):1291–307.  43. Friedman J, Karandinos G, Hart LK, Castrillo FM, Graetz N, Bourgois P. Structural vulnerability to narcotics-driven firearm violence: An ethnographic and epidemiological study of Philadelphia’s Puerto Rican inner-city. PLoS ONE 2019;14(11):1–25.  44. Wilmsen C, de Castro AB, Bush D HM. System Failure: Work Organization and Injury Outcomes among Latino Forest Workers. J Agromedicine 2019;24(2):186–96.  45. Allen ST, White RH, Grieb SM, Kilkenny E, Sherman SG, Hopkins J. Take-home naloxone possession among people who inject drugs in rural West Virginia. Drug Alcohol Dependence 2019;204:1–15.  46. Wilmsen C, Bush D B-AD. Working in the Shadows: Safety and Health in Forestry Services in Southern Oregon. J forest 2015;113(3):315–24.  47. Lavalley J, Kastor S, Tourangeau M, Goodman A, Kerr T. You just have to have other models, our DNA is different: The experiences of indigenous people who use illicit drugs and/or alcohol accessing substance use treatment. Harm Reduction Journal 2020;17(1):1–10. |
| Inclusion of Study Population | Common methodological approaches included direct inclusion of members of the target population on the research team, sometimes defined as ‘expert informant’ or ‘peer researcher’, highlighting members of the research team with the lived experience, and creation of an advisory board with members of the target population | 1. Logan RI. ‘A poverty in understanding’: Assessing the structural challenges experienced by community health workers and their clients. Global Public Health 2020;15(1):137–50.  2. Andress L, Hall T, Davis S, Levine J, Cripps K, Guinn D. Addressing power dynamics in community-engaged research partnerships. Journal of Patient-Reported Outcomes 2020;4(1):0–7.  3. Silberzahn BE, Morris MB, Riegger KE, et al. Barriers and facilitators to retaining a cohort of street-based cisgender female sex workers recruited in Baltimore, Maryland, USA: Results from the SAPPHIRE study. BMC Public Health 2020;20(1):1–12.  4. Cheney AM, Newkirk C, Rodriguez K, Montez A. Inequality and health among foreign-born latinos in rural borderland communities. Social Science and Medicine 2018;215:115–22.  5. Friedman J, Syvertsen JL, Bourgois P, Bui A, Beletsky L, Pollini R. Intersectional structural vulnerability to abusive policing among people who inject drugs: A mixed methods assessment in california’s central valley. International Journal of Drug Policy 2021;87:102981.  6. Kolla G, Strike C. ‘It’s too much, I’m getting really tired of it’: Overdose response and structural vulnerabilities among harm reduction workers in community settings. International Journal of Drug Policy 2019;74:127–35.  7. Organista KC, Arreola SG, Neilands TB. La desesperación in Latino migrant day laborers and its role in alcohol and substance-related sexual risk. SSM - Population Health 2016;2:32–42.  8. Fernández-Esquer ME, Agoff MC, Leal IM. Living Sin Papeles: Undocumented Latino Workers Negotiating Life in “Illegality.” Hispanic Journal of Behavioral Sciences 2017;39(1):3–18.  9. Mayer S, Fowler A, Brohman I, et al. Motivations to initiate injectable hydromorphone and diacetylmorphine treatment: A qualitative study of patient experiences in Vancouver, Canada. International Journal of Drug Policy 2020;85:102930.  10. Duke MR. Neocolonialism and Health Care Access among Marshall Islanders in the United States. Medical Anthropology Quarterly 2017;31(3):422–39.  11. Tulimiero M, Garcia M, Rodriguez M, Cheney AM. Overcoming Barriers to Health Care Access in Rural Latino Communities: An Innovative Model in the Eastern Coachella Valley. Journal of Rural Health 2020;00:1–10.  12. Kline N. Pathogenic Policy: Immigrant Policing, Fear, and Parallel Medical Systems in the US South. Medical Anthropology: Cross Cultural Studies in Health and Illness 2017;36(4):396–410.  13. Decker MR, Marshall B, Emereson M, Kalamar A, Covarrubias L, Astone N, Wang Z GE. Respondent-driven sampling for an adolescent health study in vulnerable urban settings: a multi-country study. Journal of Adolscent Health 2014;55(6 0):1–17.  14. Organista KC, Worby PA, Quesada J, Arreola SG, Kral AH KS. Sexual Health of Latino Migrant Day Labourers under Conditions of Structural Vulnerability. Culture, Health and Sexuality 2013;15(1):1–14.  15. Glick JL, Lim S, Lim S, et al. Structural vulnerabilities and HIV risk among sexual minority female sex workers (SM-FSW) by identity and behavior in Baltimore, MD. Harm Reduction Journal 2020;17(1):1–9.  16. Stuesse A. When They’re Done with You: Legal Violence and Structural Vulnerability among Injured Immigrant Poultry Workers. Anthropology of Work Review 2018;39(2):79–93.  17. Wilmsen C, Bush D B-AD. Working in the Shadows: Safety and Health in Forestry Services in Southern Oregon. J forest 2015;113(3):315–24.  18. Lavalley J, Kastor S, Tourangeau M, Goodman A, Kerr T. You just have to have other models, our DNA is different: The experiences of indigenous people who use illicit drugs and/or alcohol accessing substance use treatment. Harm Reduction Journal 2020;17(1):1–10. |
